# Low Rates of Diagnosis and Treatment of Iron Deficiency Anemia After an Acute Severe Gastrointestinal Hemorrhage

**DOI:** 10.21203/rs.3.rs-5307617/v1

**Published:** 2024-11-11

**Authors:** Elissa S. Lin, Usah Dutson, Dennis M. Jensen

**Affiliations:** David Geffen School of Medicine at UCLA; VA GI Hemostasis Research Unit at West Los Angeles VA Medical Center; David Geffen School of Medicine at UCLA

**Keywords:** Iron deficiency anemia, Severe GI bleeding

## Abstract

**Background:**

Few studies report about management of iron deficiency anemia after a severe, acute gastrointestinal bleed. Most include good risk patients with upper gastrointestinal bleeds and report only laboratory improvements but not clinical outcomes.

**Aims:**

To assess management of iron deficiency anemia and clinical outcomes of patients after a severe, acute gastrointestinal bleed from either upper or lower sources in an unselected group of patients.

**Methods.:**

Retrospective analysis of adult patients hospitalized with severe gastrointestinal bleeding in two referral centers. They had endoscopic diagnoses of lesions including non-variceal upper, variceal, and lower sites (diverticulosis or other colon sources). Analyses were of rates of iron studies ordered and iron treatments up to 4 months post discharge. Composite clinical outcomes were also assessed and analyzed.

**Results:**

For 337 patients studied, iron studies were ordered in only 50%. When tested, iron deficiency was diagnosed in 75% of anemias. Intravenous iron or oral iron was prescribed in only 7.1% and 26.7% of patients respectively. By 4 months, 94% of patients treated with intravenous iron and 80% treated with oral iron achieved ≥ 2 g/dL increase in hemoglobin level. Patients with high rates of severe comorbidities and severe anemia had poorer clinical outcomes than others with severe anemia and less comorbidity.

**Conclusions:**

Despite significant anemia after a severe gastrointestinal bleed from common diagnoses, iron studies were not routinely ordered. Iron deficiency anemia was infrequently recognized or treated with iron therapies. Patients with severe co-morbidities and anemia after an acute gastrointestinal bleed had poor clinical outcomes.

## 1. Introduction

Iron deficiency anemia (IDA) is one of the leading causes of years lived with disability and often develops after acute severe gastrointestinal bleeding (GIB) [1]. Despite the known negative effects of IDA, IDA is often currently underdiagnosed and underrecognized in acute GIB populations [2]. This may relate to reports that RBC transfusions for hemoglobin of more than seven grams resulted in significantly higher mortality rates of patients with severe UGI hemorrhage, especially in cirrhotic patients with variceal hemorrhage [3]. Since that report, patients with severe anemia after an IDA from all types of acute severe GI hemorrhage are not transfused as in the past and usually leave the hospital with severe, untreated IDA [4,5].

While a recent systematic review from Portugal about the diagnosis and treatment of IDA in GI bleeding summarized results on improvements in laboratory parameters with iron therapy, the authors did not include results about improvements or worsening of clinical outcomes of patients [2]. That is because there are no studies reporting that major clinical outcomes are significantly improved by treatment of severe IDA after a severe GI hemorrhage.

Acute GIB can be associated with significant morbidity and mortality. A retrospective cohort study of 2238 patients admitted for non-variceal upper GIB reported that 30-day mortality was 4.9%, 1-year mortality was 13.9%, and 2-year mortality was 19.5% [6]. Re-bleeding within 30 days was observed in 116 (4.9%) of patients [6]. Patients with IDA may have various clinical symptoms including fatigue and weakness, poor concentration, shortness of breath, and decreased exercise tolerance and complications related to worsening of major co-morbidities [7, 8].

In patients with untreated severe IDA, a correlation has been reported with poor outcomes in cardiac and abdominal surgery post-operatively [9]. In contrast, recent reports about severe anemia after acute GI bleeds (e.g. clinical guidelines and clinical practice updates) include recommendations about laboratory diagnoses and iron therapies for IDA, but do not include study results about whether iron treatment improves or worsens major clinical outcomes of patients especially those with major co-morbidities [2, 11, 12].

Although the potential role of IDA in worsening clinical outcomes has been described, there are very few reports about IDA actually worsening outcomes after severe acute GI bleeds and none about whether early treatment with iron improves major clinical outcomes. Also very few results are reported about the prevalence, risk factors for complications, and best management strategies of patients with IDA after common causes of severe GI hemorrhage other than peptic ulcers. That includes lack of results and reports in other common UGI and LGI sources of hemorrhage such as diverticulosis, which are prevalent in hospitals.

The specific aims of this study of severe IDA after severe acute GI bleeds were to report the rates of testing for and diagnosis of iron deficiency anemia, rates and types of treatment of IDA, and major clinical outcomes and complications from both common UGI and LGI sources. Our hypothesis was that patients with severe acute GIB frequently have severe IDA which is not being treated very often and therefore complications of severe co-morbidity can result which are potentially preventable.

## 2. Materials and Methods

### Study Design

This is a retrospective study of prospectively collected data of patients from the University of California Los Angeles (UCLA) Medical Center and the Veterans Administration Greater Los Angeles Healthcare System. Patients were identified through the Center for Ulcer Research and Education (CURE) Hemostasis Research Unit databases and through the UCLA Clinical and Translational Science Institute. All patients were previously enrolled in Institutional Review Board (IRB) approved studies of severe GI hemorrhage at the two centers. This secondary data analysis study of IDA was also IRB approved. Data were retrieved from electronic databases and missing results from medical records at each center including Epic electronic record systems (Epic Systems Corporation, WI, USA) and Computerized Patient Record System – CPRS (Department of Veterans Affairs, USA). Data analyzed in this study were collected from 2010 to 2023.

### Inclusion and Exclusion Criteria

Inclusion criteria were adult patients aged 18 years or older who were hospitalized for a severe acute GIB due to one of the following diagnostic sources: non-variceal upper GIB, esophageal or gastric varices, diverticular bleeding, and other lower GIB sources including delayed post-polypectomy induced ulcers (PPIU) bleeding, internal hemorrhoids, angiomas, rectal ulcers and ischemic colitis.

Patients were excluded if they had another endoscopic diagnosis (e.g. inflammatory bowel disease-IBD, GI neoplasms, erosions, or small bowel lesions). Also excluded from this study were patients with chronic anemia because of severe renal failure on dialysis and other types of chronic anemia (e.g. hemolytic anemia, sideroblastic anemia, or thalassemia). In addition patients were excluded who had rebleeding within 2 months of discharge, death before 2 months of follow up, and loss to follow up within 4 months of discharge.

### Definitions

Severe acute GIB was defined as having clinical signs of overt GI bleeding (hematemesis, hematochezia, and/or melena) and a decrease in hemoglobin of ≥ 2 grams per deciliter (g/dl) from baseline. IDA was defined as serum iron < 50 mcg/dL, ferritin < 30 ng/mL, or a transferrin saturation < 20%. Patients were categorized into three treatment groups after the acute GI bleed: intravenous (IV) iron, oral iron, and no iron supplementation or recommendation for dietary iron only.

#### Data Collection

Data were collected on standard forms designed by the investigators. These included demographics, laboratory and endoscopic results, types of iron treatments, and major outcomes (e.g. emergency room and clinic visits, repeat hospitalizations, red blood cell (RBC) transfusions, worsening of co-morbidities, adverse events of treatments and deaths), We also recorded rates ordering iron studies, diagnosing IDA, and treatment of IDA. Results were collected up to 4 months after discharge.

#### Outcome Measures and Comparisons

The primary outcomes analyzed were rates that iron studies were ordered on patients admitted with acute severe GIB during hospitalization and in follow up, rates of IDA diagnosis, and rates of iron treatment. As a clinical outcome measure of major events, we utilized a composite clinical outcomes score [10]. A score of 1 point was given for each of the following: emergency department (ER) visits, RBC transfusions, worsening of major comorbidities, major complication of treatment, or hospital readmission. A score of 6 points was given for death. We also stratified composite score outcomes by GIB diagnostic subgroups and by acute anemia versus acute on chronic anemia on initial hospitalization. Additional outcomes included hemoglobin trends during the follow up, rates of GIB recurrence, adverse events associated with iron supplementation, and provider specialty ordering the iron studies.

#### Statistical Analysis

The Excel version 16.77.1 (Microsoft Corporation, Redmond, WA) was used for data collection. The RStudio Version 2023.12.1 + 402 was utilized for data management and analysis. Quantile-quantile plots and Shapiro-Wilk tests were employed to assess data distribution and normality. Results were reported according to their distribution, number of cases (n), and medians with interquartile ranges (IQR). Additionally, for composite outcomes score analyses, means with standard deviations and confidence intervals are provided. Statistical comparisons were performed using Pearson’s Chi-squared test for categorical data and Kruskal-Wallis rank sum test for continuous data, with Fisher’s exact test applied where appropriate. To account for multiple comparisons, Bonferroni correction was implemented for post-hoc analyses. Composite outcomes score analyses included comparisons of composite scores among the three iron treatment groups using Kruskal-Wallis rank sum test and between iron treatment subgroups within each gastrointestinal bleeding subgroup using Wilcoxon rank sum test. A p-value of 0.05 was considered statistically significant.

## 3. Results

### Baseline Patient Characteristics

506 cases were reviewed for the CURE databases and 337 patients met the inclusion criteria and formed the study population. Of those, 24 patients were treated with IV iron, 90 patients were treated with oral iron, and 223 patients were not treated with iron. Demographics and clinical characteristics of the study population are shown in Table 1. 73% of patients were male and 53% were white. The mean age of the patients was 64 years and there was no significant difference among iron treatment groups.

246 (73%) of patients presented with acute anemia and 91 (27%) with acute on chronic anemia. In terms of co-morbidities, patients treated with IV iron had significantly higher rates of peripheral vascular disease and acute kidney injury. They also had higher rates of other comorbidities with the exception of liver disease and major infections.

### Diagnosis and Treatment Rates of Iron Deficiency Anemia

**Figure 1.**
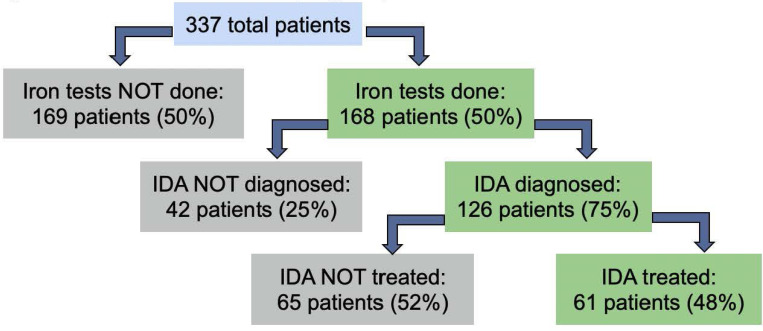
Rates of iron studies ordered, diagnosis, and treatment.

Iron tests were performed in only 168 (50%) of patients. 35% of patients had iron studies performed during hospitalization, 30% had iron testing in the 4-month follow up period, and 15% had iron testing performed both during hospitalization and in follow up. For the 168 patients with iron studies performed, IDA was diagnosed in 75% − 126 patients. For those patients with a diagnosis of IDA, only 61 (48%) received either IV or oral iron. For patients with documented IDA, 52% did not receive iron treatment (see Fig. 1).

The majority of patients who were treated with iron during hospitalization were also prescribed iron treatment at discharge or in follow up (94%). Among patients who were untreated during their hospitalization, only 2 patients (0.9%) were prescribed iron treatment at discharge or in the follow up period.

During hospitalization and in follow up, the majority of iron tests were ordered by primary care or internal medicine physicians (72% and 81% respectively). For patients with iron tests ordered during follow up and with IDA diagnosed, ferritin normalized within 4 months in 80% of patients receiving IV iron vs. 64% of patients receiving oral iron vs. 59% of patients who were not prescribed iron supplementation (p = 0.33). Transferrin saturation (TSAT) normalized in 56% of patients receiving IV iron vs. 45% of patients receiving oral iron vs. 23% of patients who were not prescribed iron supplementation (p = 0.02). Refer to Table 2 for details of iron study results.

### Hemoglobin and Transfusion Outcomes

Patients had a baseline median hemoglobin of 13 g/dL before the acute GIB which decreased to a median 7.9 g/dL after the acute GIB. The median hemoglobin on discharge was 9.7 g/dL. Patients were transfused a mean of 2 units of RBC’s during hospitalization. Only 72% of patients had hemoglobin levels ordered during follow up. For those patients, hemoglobin normalized to baseline values in 62% of patients treated with IV iron, 36% of patients treated with oral iron supplementation, and 24% of patients who were not prescribed iron supplementation. Refer to Table 2 for details on hemoglobin levels.

### Composite Score Analysis and Adverse Events

Patients who received iron treatment had higher rates of severe co-morbidities and during follow-up had overall clinical worsening and higher (worse) clinical outcome scores than patients in the diet/no treatment group. However, these were not statistically significantly different. Major clinical outcomes in all patients included ER visits in 11%, recurrent GIB in 11%, and transfusion of RBC’s in 12% of patients. There were no significant differences in composite outcome scores [10] among study groups, although there was a trend toward higher composite outcome scores in the IV iron group. Patients with severe co-morbidities had significant higher rates of worsening comorbidities during follow-up. This was inspite of the type of iron treatment or no iron treatment. There was only 1 death between 2 and 4 months and this occurred in the untreated group due to esophageal varices rebleeding.

We also compared outcome scores in patients with acute anemia to patients with acute on chronic anemia. We found a significantly higher rate of worse clinical outcomes in the acute on chronic anemia subgroup compared to the acute anemia cohort (45% vs 27%, p = 0.001). This is despite similar rates of IDA diagnosed and similar rates of iron treatment. We also assessed clinical outcomes for different GIB subgroups and compared composite clinical outcome scores for patients treated with either IV or oral supplementation versus no iron treatment. We found no significant differences in composite clinical score outcomes. See Table 3 for composite score analyses and Table 4 for a breakdown of clinical adverse events.

### Side Effects of Iron Therapy

Side effects of iron therapy were minimal. In the IV iron group, 1 (4.5%) patient reported constipation and this resulted in a reduction of the IV iron dose. In the oral iron group, 3 patients (3.5%) reported side effects such as lightheadedness, nausea or vomiting, and constipation. Iron was discontinued in one patient who received oral iron supplementation. No adverse events were reported in the diet/no treatment group. Iron related side effects are detailed in Table 4.

## 4. Discussion

Our retrospective, observational study is one of the few to highlight major gaps in iron deficiency testing and treatment in patients with major co-morbidities after an acute severe GIB. Our analysis included patients with the most common diagnoses for acute severe GIB from both the upper and lower GI tracks. Major clinical outcomes were assessed for four months after discharge and reported with a new clinical outcomes composite score [10]. Despite significant anemia after acute severe GIBs, iron studies were not routinely ordered and iron deficiency was infrequently evaluated or treated during hospitalization or in follow up. Of the patients for whom iron studies were ordered, there was a high prevalence of IDA (e.g. 75%) but only 48% of those with IDA were treated with supplemental iron. IV iron was prescribed the least.

Patients treated with IV iron had worse baseline co-morbidity profiles compared to the other treatment groups. High risk patients in any subgroup had the highest rate of worsening of comorbidities during follow up. Patients with acute on chronic anemia also had significantly worse composite outcome scores compared to patients with acute anemia despite having similar rates of diagnosis and treatment of IDA. For individual subgroups of specific sources of GIB, there were no significant differences in composite outcome scores when comparing iron treatment to no treatment. However in this observational study, co-morbidities were not balanced at baseline as would be possible in an RCT.

A few observational studies from other countries reported that iron therapy is under-prescribed in patients with anemia after an acute GIB. In the retrospective study by El-Halabi et al [4], only 23 (71/307) of patients admitted to the hospital with an acute GIB received iron therapy during hospitalization. On hospital discharge, only 22% of patients had instructions in their discharge summary to check iron studies in the outpatient setting. In another retrospective study of acute upper GIB by Bager and Dahlerup^5^, oral supplementation was recommended for only 16% of patients with anemia on discharge. Our USA study adds to the existing evidence that for patients admitted with common upper and lower sources of severe acute GIB, the rates of iron studies ordered for diagnosis of IDA are low.

There have been three randomized controlled trials (RCT) from other countries published comparing the efficacy of IV versus oral iron supplementation following severe acute GIB. In 2014, Bager and Dahlerup^13^ reported a Danish RCT of 91 patients with IDA following acute upper GIBs. Patients were randomized to a 3-month regimen of oral iron (100mg ferrous sulfate twice daily), a single administration of IV ferric carboxymaltose (FCM), or placebo. At the end of the trial, 70% of the placebo group remained anemic compared to 17% of the iron treatment groups (p < 0.01). The second RCT was published in 2019 by Ferrer-Barceló et al [14]. comparing FCM to oral ferrous sulfate in patients with anemia secondary to non-variceal upper GIB. After 42 days, normalization of hemoglobin was achieved by 100% of the FCM treated patients compared to 61.3% of the oral iron treated patients (p < 0.001). The third RCT by Tabish et al [15]. reported results of patients with an acute esophageal variceal hemorrhage who were randomized to either IV FCM or oral iron. Anemia significantly improved with IV iron compared to oral iron. However, there were no significant differences in liver-related outcomes nor other clinical outcomes during follow-up [15].

There are several important limitations of the reported studies when considering the available evidence about iron therapy in the acute GI bleed population. First, all the RCT’s originated from outside the United States (e.g. Europe, India) and most excluded high-risk patients such as those with significant cardiovascular, pulmonary, and kidney diseases, except the study of anemia after variceal hemorrhage [15]. High-risk patients were included in our study. Second, most studies were funded by pharmaceutical companies which market to IV iron products which were studied. This can lead to potential bias towards the use of certain intravenous iron formulations. In contrast, our study was funded by non-pharmaceutical sources. Third, none of these studies reported about changes or improvements in major clinical outcomes, except the Tabish Study [15]. Tabish reported laboratory improvement in anemia with IV iron in cirrhotic patients after a severe esophageal variceal hemorrhage but no improvement in clinical outcomes [15]. We report major clinical outcomes in our study. Finally, most of the study populations only included patients with upper GI bleeds and not LGI bleeds, which are increasing in prevalence in elderly patients and IDA is common.

A recent American Gastroenterologic Association (AGA) guideline and a clinical practice update do not report about the evaluation and management of IDA after an acute severe GIB [11, 12]. Both the 2020 AGA guideline and the 2024 clinical practice update focused on iron deficiency anemia in uncomplicated patients with IDA. They recommended an initial trial of iron supplementation and not routine use of endoscopy [11, 12]. There were no recommendations nor results presented about the evaluation of IDA in patients with acute severe GIB, nor clinical outcomes with treatment of IDA. Our study highlights that a large proportion of high-risk patients have IDA after acute severe GIB and these are the patients who could potentially benefit the most from early treatment of IDA. That is by preventing complications of severe IDA, improving symptoms, and potentially reducing rehospitalizations and transfusions of RBC’s during follow-up.

### Limitations and recommendations

Our study has several limitations. It was an observational study. It was not an interventional study nor an RCT. The majority of our patient cohort was male (73%) and white (53%) which may limit the generalizability of our results to other populations. Baseline differences in co-morbidities were assessed but not balanced. Current actively and severity of major co-morbidities were not assessable in this retrospective study. Because of this imbalance and the small groups of patients especially in the IV iron group, a multivariate analysis of risk factors could not be performed. However, our results expand the understanding about the current prevalence of IDA, treatment patterns, and clinical outcomes for different acute GI bleeds in the USA. Without an RCT which balances baseline co-morbidities, final conclusions can not be drawn about whether iron therapy significantly improves major clinical outcomes after an acute, severe GIB. A well designed RCT is needed for such an assessment, especially in patients after a severe non-variceal UGI and LGI hemorrhages. Based upon Tabish’s report that patients with cirrhosis and esophageal variceal bleeds did not have improvements in clinical outcomes [15], we would not recommend including that subgroup in such RCTs. Additionally, certain clinical outcomes were not assessed in our study. These are patient reported fatigue, energy, and quality of life. Nor are cost analyses reported here or in any prior report. Along with improvements in laboratory parameters (e.g. hemoglobin and iron studies), these are important measures to analyze and report in future RCTs. With the limitations in reports to date, we are in agreement with a recent narrative analysis and review about the need for more results and further prospective studies in patients with IDA after acute severe GI bleeds [16].

## Conclusions

Several important knowledge gaps exist in our understanding about the best management of IDA after a severe acute GIB. Recent studies on the efficacy of iron treatments and guidelines have primarily focused on low risk patients without major comorbidities and improvements in laboratory parameters of IDA. Yet in our report patients with major comorbidities experience worse clinical outcomes related to IDA. We report low rates of iron studies ordered and high rates of chronic iron deficiency after severe acute GIBs. We also report that patients with severe co-morbidities and severe anemia after a GIB had poor clinical outcomes. Prospective, randomized studies comparing oral and intravenous iron treatments are needed to assess the clinical outcomes of high-risk patients, balanced by their severe co-morbidities and diagnostic subgroups. Except for the patients with cirrhosis and esophageal variceal bleeds, this is the large group of patients who are expected to potentially benefit most from early treatment of IDA after a severe GI hemorrhage.

## Figures and Tables

**Table 1:** Cohort characteristics and patient demographics.

	IV Iron N = 24 (7.1%)	Oral Iron N = 90 (26.7%)	Diet/No treatment N = 223 (66.2%)	Overall N = 337	P-value
*GIB Groups*					
Non-variceal upper GIB	11 (46%)	33 (37%)	72 (32%)	116 (34%)	
Diverticular bleed	6 (25%)	25 (28%)	61 (27%)	92 (27%)	
Other lower GIB	6 (25%)	21 (23%)	39 (17%)	66 (20%)	
Gastroesophageal varices	1 (4.2%)	11 (12%)	51 (23%)	63 (19%)	
*Demographics*					
Age in years	71 (63, 75)	66 (55, 76)	63 (54, 74)	64 (54,75)	0.130
Sex at birth					0.752
Male	18 (75%)	63 (70%)	165 (74%)	246 (73%)	
Female	6 (25%)	27 (30%)	58 (26%)	91 (27%)	
*Race and Ethnicity*					0.842
White/ Caucasian	11 (46%)	43 (48%)	125 (56%)	179 (53%)	
Black/ African American	5 (21%)	21 (23%)	37 (17%)	63 (19%)	
Hispanic	4 (17%)	12 (13%)	29 (13%)	45 (13%)	
Asian	2 (8.3%)	10 (11%)	21 (9.4%)	33 (9.8%)	
Other	0 (0%)	1 (1.1%)	2 (0.9%)	3 (0.9%)	
Native American Indian	2 (8.3%)	3 (3.3%)	9 (4.0%)	14 (4.2%)	
*Anemia type*					0.346
Acute anemia	20 (83%)	68 (76%)	158 (71%)	246 (73%)	
Acute on chronic anemia	4 (17%)	22 (24%)	65 (29%)	91 (27%)	
*Comorbidities*					
Cardiovascular disease	9 (38%)	38 (42%)	69 (31%)	116 (34%)	0.155
Pulmonary disease	4 (17%)	13 (14%)	26 (12%)	43 (13%)	0.591
Chronic kidney disease	2 (8.3%)	11 (12%)	15 (6.7%)	28 (8.3%)	0.254
Acute kidney injury	4 (17%)	12 (13%)	11 (4.9%)	27 (8.0%)	0.009
Liver disease	7 (29%)	23 (26%)	66 (30%)	96 (28%)	0.771
Peripheral vascular disease	3 (13%)	4 (4.4%)	4 (1.8%)	11 (3.3%)	0.019
Infection	0 (0%)	2 (2.2%)	8 (3.6%)	10 (3.0%)	0.872

Data are presented as median (interquartile range) or number (percentage).

**Table 2:** Iron studies and changes in hemoglobin during hospitalization and in follow up

	IV Iron N = 24 (7.1%)	Oral Iron N = 90 (26.7%)	Diet/No treatment N = 223 (66.2%)	Overall N = 337	P- value
*During Hospitalization*					
Index iron tests done (during hospitalization)	19 (79%)	39 (43%)	61 (27%)	119 (35%)	<0.001
Index ferritin	52 (16, 165)	44 (25, 85)	60 (23, 184)	48 (23, 166)	0.649
Index iron	37 (22, 69)	28 (16, 70)	45 (30, 69)	41 (22, 69)	0.367
Baseline hemoglobin (g/dL)	12.00 (11.20, 13.83)	12.60 (11.60, 14.00)	13.10 (12.00, 14.00)	13.00 (11.70, 14.00)	0.130
Lowest hemoglobin level	7.30 (6.30, 7.93)	7.60 (6.43, 8.58)	8.20 (7.15, 9.25)	7.90 (6.90, 9.00)	<0.001
Hemoglobin level at discharge	9.05 (8.48, 9.63)	9.35 (8.60, 10.08)	10.00 (9.05, 10.90)	9.70 (8.90, 10.60)	<0.001
*4 Month Follow Up*					
Iron tests done during follow-up	16 (67%)	36 (40%)	50 (22%)	102 (30%)	<0.001
Follow-up ferritin	109 (69, 288)	79 (19, 137)	40 (14, 93)	61 (19, 137)	0.045
Follow-up iron	64 (50, 73)	44 (29, 86)	45 (32, 65)	50 (32, 76)	0.219
Follow-up TIBC	313 (269, 382)	338 (280, 372)	366 (311, 417)	341 (286, 396)	0.077
Follow-up TSAT	21 (16, 27)	16 (8, 25)	14 (8, 19)	16 (8, 23)	0.056
Ferritin Normalized (> = 30)	12 (80%)	16 (64%)	24 (59%)	52 (64%)	0.333
TSAT Normalized (> = 20)	9 (56%)	15 (45%)	11 (23%)	35 (36%)	0.021
Hemoglobin checked during follow-up	21 (88%)	70 (78%)	153 (69%)	244 (72%)	0.059
Hemoglobin level at 1 month	10.90 (10.00, 11.70)	10.35 (9.20, 11.50)	11.00 (9.70, 11.90)	10.80 (9.60, 11.80)	0.248
Hemoglobin level at 2 months	12.10 (10.90, 12.70)	11.30 (9.30, 12.60)	11.10 (9.88, 12.30)	11.35 (9.95, 12.48)	0.245
Hemoglobin level at 3 months	11.50 (10.58, 12.58)	10.10 (8.70, 10.80)	11.20 (9.70, 12.20)	10.85 (9.60, 12.20)	0.031
Hemoglobin level at 4 months	11.80 (9.40, 12.55)	11.70 (9.48, 13.35)	11.10 (9.90, 12.30)	11.25 (9.60, 12.43)	0.750
Hemoglobin normalized to baseline	13 (62%)	25 (36%)	37 (24%)	75 (31%)	0.001

Data are presented as median (interquartile range) or number (percentage).

**Table 3:** Composite clinical outcome score analyses

	IV Iron N = 24 (7.1%)	Oral Iron N = 90 (26.7%)	Diet/No treatment N = 223 (66.2%)	Overall N = 337	P-value
*Composite Score*					0.073
Mean (SD)	1.13 (1.62)	0.90 (1.32)	0.67 (1.30)		
Median (IQR)	0.00 (0.00, 2.00)	0.00 (0.00, 2.00)	0.00 (0.00, 1.00)		
Range	0.00, 5.00	0.00, 4.00	0.00, 9.00		
	**Non-Variceal Upper GIB**				
	Treated with Iron (IV/Oral) N = 39 (37%)		No Iron Treatment N = 66 (63%)		
*Composite Score*					0.7
Mean (SD)	0.85 (1.29)		0.74 (1.19)		
Median (IQR)	0.00 (0.00, 2.00)		0.00 (0.00, 1.00)		
Range	0.00, 5.00		0.00, 4.00		
	**Diverticular Bleeding**				
	Treated with Iron (IV/Oral) N = 31 (34%)		No Iron Treatment N = 61 (66%)		
*Composite Score*					0.6
Mean (SD)	0.35 (0.75)		0.31 (0.74)		
Median (IQR)	0.00 (0.00, 0.00)		0.00 (0.00, 0.00)		
Range	0.00, 3.00		0.00, 3.00		
	**Varices or Portal Hypertensive Lesions**				
	Treated with Iron (IV/Oral) N = 13 (19%)		No Iron Treatment N = 56 (81%)		
*Composite Score*					0.3
Mean (SD)	1.54 (1.56)		1.14 (1.81)		
Median (IQR)	2.00 (0.00, 3.00)		0.00 (0.00, 2.00)		
Range	0.00, 4.00		0.00, 9.00		
	**Angioma Syndromes**				
	Treated with Iron (IV/Oral) N = 13 (59%)		No Iron Treatment N = 9 (41%)		
*Composite Score*					0.4
Mean (SD)	2.08 (2.06)		1.00 (1.32)		
Median (IQR)	3.00 (0.00, 4.00)		1.00 (0.00, 1.00)		
Range	0.00, 5.00		0.00, 4.00		
	**Acute Anemia N = 246 (73%)**		**Acute on Chronic Anemia N = 91 (27%)**		
*Composite Score*					<0.001
Mean (SD)	0.56 (1.06)		1.32 (1.78)		
Median (IQR)	0.00 (0.00, 1.00)		0.00 (0.00, 3.00)		
Range	0.00, 5.00		0.00, 9.00		

Abbreviations: SD = standard deviation; IQR = interquartile range

**Table 4:** Adverse Outcome Events by Type of Iron Supplementation

	IV Iron N = 24 (7.1%)	Oral Iron N = 90 (26.7%)	Diet/No treatment N = 223 (66.2%)	Overall N = 337	P- value
ER visit	4 (17%)	7 (7.8%)	25 (11%)	36 (11%)	0.376
Number of ER visits	1.00 (1.00, 1.00)	2.00 (1.00, 2.00)	1.00 (1.00, 1.25)	1.00 (1.00, 2.00)	0.121
GIB during follow-up	4 (17%)	14 (16%)	20 (9.0%)	38 (11%)	0.148
Number of GIB episodes	1.00 (1.00, 1.25)	1.00 (1.00, 2.00)	1.00 (1.00, 1.00)	1.00 (1.00, 1.00)	0.562
Readmission	6 (25%)	24 (27%)	45 (20%)	75 (22%)	0.433
Number of readmissions	1.50 (1.00, 2.75)	1.00 (1.00, 2.00)	1.00 (1.00, 2.00)	1.00 (1.00, 2.00)	0.682
Worsening comorbidities	8 (33%)	19 (21%)	31 (14%)	58 (17%)	0.031
Cardiovascular disease complications	3 (13%)	7 (7.8%)	7 (3.1%)	17 (5.1%)	0.033
Pulmonary disease complications	3 (16%)	2 (2.6%)	3 (1.4%)	8 (2.6%)	0.007
Chronic kidney disease complications	0 (0%)	2 (2.2%)	2 (0.9%)	4 (1.2%)	0.499
Acute kidney injury complications	2 (8.3%)	5 (5.6%)	3 (1.3%)	10 (3.0%)	0.018
Liver disease complications	1 (4.2%)	4 (4.4%)	14 (6.3%)	19 (5.6%)	0.856
Peripheral vascular disease complications	0 (0%)	0 (0%)	1 (0.4%)	1 (0.3%)	>0.999
Infection complications	0 (0%)	5 (5.6%)	6 (2.7%)	11 (3.3%)	0.426
*Iron Related Side Effects*					>0.999
Constipation	1 (100%)	1 (33%)	0 (NA%)		
Lightheadedness	0 (0%)	1 (33%)	0 (NA%)		
NV	0 (0%)	1 (33%)	0 (NA%)		

Data are presented as median (interquartile range) or number (percentage).

## References

[1] Global, regional, and national incidence, prevalence, and years lived with disability for 328 diseases and injuries for 195 countries, 1990–2016: a systematic analysis for the Global Burden of Disease Study 2016. Lancet (London, England) 2017; 390(10100): 1211–59. 28919117 10.1016/S0140-6736(17)32154-2 PMC5605509

[2] Cotter J, Baldaia C, Ferreira M, Macedo G, Pedroto I. Diagnosis and treatment of iron-deficiency anemia in gastrointestinal bleeding: A systematic review. World J Gastroenterol 2020; 26(45): 7242–57. 33362380 10.3748/wjg.v26.i45.7242 PMC7723662

[3] Villanueva C, Colomo A, Bosch A, Transfusion strategies for acute upper gastrointestinal bleeding. N Engl J Med 2013; 368(1): 11–21. 23281973 10.1056/NEJMoa1211801

[4] El-Halabi MM, Green MS, Jones C, Salyers WJ, Jr. Under-diagnosing and under-treating iron deficiency in hospitalized patients with gastrointestinal bleeding. World J Gastrointest Pharmacol Ther 2016; 7(1): 139–44. 10.4292/wjgpt.v7.i1.139 PMC4734947 26855820

[5] Bager P, Dahlerup JF. Lack of follow-up of anaemia after discharge from an upper gastrointestinal bleeding centre. Dan Med J 2013; 60(3): A4583. 23484606

[6] Subramaniam K, Spilsbury K, Ayonrinde OT, Red blood cell transfusion is associated with further bleeding and fresh-frozen plasma with mortality in nonvariceal upper gastrointestinal bleeding. Transfusion 2016; 56(4): 816–26. 26718025 10.1111/trf.13446

[7] Kim YJ, Han KD, Cho KH, Kim YH, Park YG. Anemia and health-related quality of life in South Korea: data from the Korean national health and nutrition examination survey 2008–2016. BMC Public Health 2019; 19(1): 735–42. 31196013 10.1186/s12889-019-6930-y PMC6567528

[8] Camaschella C. Iron-deficiency anemia. N Engl J Med 2015; 372(19): 1832–43. 25946282 10.1056/NEJMra1401038

[9] Muñoz M, Gómez-Ramírez S, Campos A, Ruiz J, Liumbruno GM. Pre-operative anaemia: prevalence, consequences and approaches to management. Blood Transfus 2015; 13(3): 370–9. 26192787 10.2450/2015.0014-15 PMC4614288

[10] Jensen DM, Barkun A, Cave D, Gralnek IM, Jutabha R, Laine L, Lau JYW, Saltzman JR, Soetikno R, Sung JJY. Acute gastrointestinal bleeding: proposed study outcomes for new randomised controlled trials. Aliment Pharmacol Ther. 2021;54(5):616–626. 34288017 10.1111/apt.16483 PMC9385213

[11] Ko CW, Siddique SM, Patel A, AGA Clinical Practice Guidelines on the Gastrointestinal Evaluation of Iron Deficiency Anemia. Gastroenterology 2020; 159(3): 1085–94. 32810434 10.1053/j.gastro.2020.06.046

[12] DeLoughery TG, Jackson CS, Ko CW, Rockey DC. AGA Clinical Practice Update on Management of Iron Deficiency Anemia: Expert Review. Clin Gastroenterol Hepatol 2024; 22(8):1575–1583. 38864796 10.1016/j.cgh.2024.03.046

[13] Bager P, Dahlerup JF. Randomised clinical trial: oral vs. intravenous iron after upper gastrointestinal haemorrhage--a placebo-controlled study. Aliment Pharmacol Ther 2014; 39(2): 176–87. 24251969 10.1111/apt.12556

[14] Ferrer-Barceló L, Sanchis Artero L, Sempere García-Argüelles J, Randomised clinical trial: intravenous vs oral iron for the treatment of anaemia after acute gastrointestinal bleeding. Aliment Pharmacol Ther 2019; 50(3): 258–68. 31197861 10.1111/apt.15327 PMC6771644

[15] Tabish M, Agarwal S, Gopi S, Rana R, Ahmed S, Gunjan D, Sharma S, Saraya A. Randomized Controlled Trial of Intravenous Ferric Carboxymaltose vs Oral Iron to Treat Iron Deficiency Anemia After Variceal Bleed in Patients With Cirrhosis. Am J Gastroenterol 2024; 119(10):2061–2069. 38517084 10.14309/ajg.0000000000002775

[16] Lanas A, Andrews JM, Lau J, Toruner M, Bromley SE, Gralnek IM. Management of iron-deficiency anemia following acute gastrointestinal hemorrhage: A narrative analysis and review. J Gastroenterol Hepatol 2023; 38(1):23–33. 36266733 10.1111/jgh.16033

